# Microsatellite Stability in STR Analysis *Aspergillus fumigatus* Depends on Number of Repeat Units

**DOI:** 10.3389/fcimb.2019.00082

**Published:** 2019-03-29

**Authors:** Theun de Groot, Jacques F. Meis

**Affiliations:** ^1^Department of Medical Microbiology and Infectious Diseases, Canisius Wilhelmina Hospital (CWZ), Nijmegen, Netherlands; ^2^Centre of Expertise in Mycology, Radboudumc/CWZ, Nijmegen, Netherlands; ^3^Department of Medical Microbiology, Radboudumc, Nijmegen, Netherlands

**Keywords:** *Aspergillus fumigatus*, microsatellites, short tandem repeats, STR*Af*, stability

## Abstract

More than a decade ago a short tandem repeat-based typing method was developed for the fungus *Aspergillus fumigatus*. This STR*Af* assay is based on the analysis of nine short tandem repeat markers. Interpretation of this STR*Af* assay is complicated when there are only one or two differences in tandem repeat markers between isolates, as the stability of these markers is unknown. To determine the stability of these nine markers, a STR*Af* assay was performed on 73–100 successive generations of five clonally expanded *A. fumigatus* isolates. In a total of 473 generations we found five times an increase of one tandem repeat unit. Three changes were found in the trinucleotide repeat marker STR*Af* 3A, while the other two were found in the trinucleotide repeat marker STR*Af* 3C. The di- or tetranucleotide repeats were not altered. The altered STR*Af* markers 3A and 3C demonstrated the highest number of repeat units (≥50) as compared to the other markers (≤26). Altogether, we demonstrated that 7 of 9 STR*Af* markers remain stable for 473 generations and that the frequency of alterations in tandem repeats is positively correlated with the number of repeats. The potential low level instability of STR*Af* markers 3A and 3C should be taken into account when interpreting STR*Af* data during an outbreak.

## Introduction

Tandem repeats are repetitive DNA sequences of 1 or more nucleotides, which are abundantly present in eukaryotic genomes, both in coding and non-coding regions (Gemayel et al., 2010). They are characterized by their highly polymorphic nature, as the number of repeated sequences often varies within species (Genovese et al., 2018). This polymorphic nature plays an important role in adaptation toward environmental changes by affecting cellular processes like cell surface variability, plasticity in body morphology and tuning of the circadian rhythm. On the other hand it can also cause disease, like Huntington disease (Gemayel et al., 2010).

Tandem repeats are classified according to the length of their repeated sequence. Repeats consisting of 1–9 nucleotides are generally known as microsatellites, or short tandem repeats (STRs), while longer repeats are known as minisatellites (Gemayel et al., 2010). The variation in the number of tandem repeats in both micro- and minisatellites results from strand-slippage by the DNA polymerase during replication, in which a mismatch of one of more repeat units occurs after the transient dissociation of the template and nascent DNA strand. When the template strand produces a loop, one or more repeat units will be deleted from the new DNA strand, however when this occurs with the nascent strand, one or more repeat units will be inserted (Gemayel et al., 2010; Genovese et al., 2018). In addition to strand-slippage, variation in micro- and minisatellites is also caused by events during recombination, like unequal crossing over and gene conversion (Amos, 2010; Ananda et al., 2013). Tandem repeat mutation rates vary from 1:100 to 1:100.000 per locus per generation, which is at least a factor 10 more frequent than point mutations (Brinkmann et al., 1998; Legendre et al., 2007; Jansen et al., 2012; Molnar et al., 2012; Chapuis et al., 2015). This STR mutation rate is dependent on different characteristics of the tandem repeat, like the number of repeated units, unit length and repeat structure and purity (Brinkmann et al., 1998; Legendre et al., 2007; Amos, 2010).

The high variability of STRs and the relative ease of detection of these polymorphisms via PCR amplification make them ideally suited for microbial genotyping. For the fungus *A. fumigatus* a robust typing method based on nine STRs was developed (STR*Af* assay) (de Valk et al., 2005, 2007). *A. fumigatus* is the most common fungus, which can cause invasive aspergillosis in immunocompromised patients with often fatal consequences (Chowdhary et al., 2017). Previously, the high discriminatory power of the STR*Af* assay was demonstrated by distinguishing 96 genotypes within 99 presumably unrelated isolates (de Valk et al., 2005). Furthermore, its utility in epidemiological surveys was established as the epidemiological concordance among isolates from 6 outbreaks was identified (Balajee et al., 2008). Here, epidemiological related isolates with differences of only one repeat unit in a single STR marker were called micro-variation. It is unknown how to deal with these variations, since no guidelines for the interpretation of STR data have been postulated.

In order to interpret the relationship between isolates with differences in the STR markers, it is necessary to understand the dynamics of the individual markers. In a previous study we demonstrated that 30 subcultures of one *A. fumigatus* isolate did not affect any of the markers (de Valk et al., 2005). To expand our knowledge on the stability of STR markers in *A. fumigatus*, we investigated the stability of 9 highly polymorphic microsatellite markers in 100 successive generations of 5 clonally expanded *A. fumigatus* isolates with randomly chosen genotypes.

## Materials and Methods

### Generations

Four randomly chosen unrelated clinical *A. fumigatus* isolates and one reference strain (CBS 487.65) were clonally expanded up to 100 generations. All isolates were subcultured onto Sabouraud agars and incubated at 30°C until sporulation. Successive generations were grown from a single spore using the following procedure: A suspension of *A. fumigatus* spores was made in aqua dest with 0.05% Tween 40 (Merck Nederland B.V., Amsterdam, The Netherlands). The transmission of this suspensions was measured at a wavelength of 530 nm and adjusted to 80–82%, which corresponds to approximately 1.5 × 10^6^ CFU/ml. This suspension was diluted to a concentration of 3 × 10^2^ CFU/ml. About 100 μL of this suspension was plated onto a Sabouraud agar and incubated at 30°C and examined daily. A single non-sporulating colony was then picked and subcultured on a new Sabouraud agar and incubated at 30°C until sporulation. Subsequent generations were made accordingly.

### STR*Af* Assay

DNA of all generations was extracted as previously described (de Valk et al., 2005). All DNA samples were analyzed using the 9 loci of the STR*Af* panel consisting of three di-, tri-, and tetra-nucleotide repeats. PCR primers, fluorescent labels and amplification conditions were exactly as previous described (de Valk et al., 2005). Assignment of repeat numbers in each marker was performed using Fragment Profiler 1.2 software (GE Healthcare, Diegem, Belgium) and checked by visual interpretation of the electropherograms.

## Results

Four unrelated randomly chosen *A. fumigatus* isolates and one reference isolate were clonally expanded. Four isolates yielded 100 generations and one slow-sporulating isolate yielded 73 generations. In total, 473 generations were grown and analyzed with the nine STR markers of the STR*Af* assay. This yielded a total of 4,257 marker generations. Results are shown in Table 1.

**Table 1:** Overview of the marker generation of five randomly chosen isolates.

		**STR*****Af***								
**Isolate**	**Generation**	**2A**	**2B**	**2C**	**3A**	**3B**	**3C**	**4A**	**4B**	**4C**
Clinical 1	1–5	18	12	8	27	12	20	9	9	5
	6–100	18	12	8	27	12	21	9	9	5
Clinical 2	1–100	18	12	13	14	10	14	8	11	5
Clinical 3	1–36	23	23	15	39	11	50	10	26	8
	37–100	23	23	15	39	11	51	10	26	8
Clinical 4	1–5	23	19	15	53	11	7	13	9	5
	6–48	23	19	15	54	11	7	13	9	5
	49–66	23	19	15	55	11	7	13	9	5
	67–100	23	19	15	56	11	7	13	9	5
Reference	1–73	10	17	10	25	11	23	7	5	6

*Changes in number of repeats are marked in gray*.

In isolate #2 and the reference isolate no change in any of the nine STR*Af* markers was observed. In isolates #1 and #3 one alteration was found, which both took place in the STR*Af* 3C marker. After five generations of isolate #1, the number of repeat units increased from 20 to 21, while for isolate #3 it increased from 50 to 51 in the 36th generation. Most changes were observed in isolate #4, where in the 5th, 48th, and 66th generation the number of repeat units in the STR*Af* 3A marker gradually increased from 53 to 56. Altogether, five changes in the number of repeat units were observed, which all involved an increase of one repeat unit in a trinucleotide repeat marker.

## Discussion

A better understanding of the epidemiological relation between isolates can be provided by molecular fingerprinting methods. More than 10 years ago a STR-based analysis to type *A. fumigatus* (STR*Af*) was developed that consists of three times three di-, tri-, or tetra-nucleotides repeat markers (de Valk et al., 2005). In order to separate related and unrelated isolates within an outbreak, Guinea et al. suggested in 2011 to define isolates with differences in no more than 2 repeat units for a single marker as microvariants and thus genetically related, and isolates with 3 or more repeat units difference as unrelated (Guinea et al., 2011). Information regarding the stability of the STR markers of *A. fumigatus* was however rather limited. To obtain more insight in the stability of the STR*Af* markers and improve our understanding whether or not isolates are related during an outbreak, we studied the variation in repeat numbers in successive clonally expanded *A. fumigatus* isolates by subculturing single spores. In total, 473 generations were analyzed with the STR*Af* panel. In seven out of nine markers no changes were found. In STR*Af* 3A and 3C three and two mutations were found, respectively, suggesting that these repeats are potentially less stable. It is known that the variability of some tandem repeats is much higher than others (Bustamante et al., 2013) and that the number of repeat units within a STR can change very rapidly during an outbreak (Hyytiä-Trees et al., 2006; Noller et al., 2006). Importantly, the three mutations in STR*Af* marker 3A were found in one isolate, demonstrating that the former definition of microvariation for the STR*Af* assay would not have been valid for this marker if similar variations would have been found in an outbreak. The relative instability of STR*Af* marker 3A, and to a lesser extent marker 3C, has to be taken into account when interpreting the relationship between epidemiological related *A. fumigatus* isolates. Another option is the exclusion of the trinucleotide repeat panel from the STR*Af* analysis, as with the di- and tetra-nucleotide repeats only, the discriminatory power remains very high (Garcia-Rubio et al., 2018).

Four out of five alterations were found in markers with 50 or more repeat units, suggesting that mutation rate may increase with increasing repeat units. Indeed, other studies in various organisms, like primates (*Homo sapiens*), insects (*Drosophila melanogaster*), plants (*Arabidopsis Thaliana*), yeast (*Saccharomyces cerevisiae*) and bacteria (*E. coli*), have shown that the variability of repeats is mostly dependent on the number of repeat units (Wierdl et al., 1997; Vogler et al., 2006; Legendre et al., 2007). Therefore, for *A. fumigatus*, as well as for other organisms, a higher number of repeat units decreases its stability. This means that expansion of one extra repeat unit to a repeat consisting of 10 units is less likely to occur in the same period of time than the expansion of a unit to a repeat that already contain 50 units. For the calculation of relations between isolates with these variations in a marker, this means that a difference of 10 to 11 repeat units in a marker should be given more weight than a change from 50 to 51 repeat units.

All five mutations were found in the trinucleotide repeat markers STR*Af* 3A and STR*Af* 3C, while no mutations were present in the di- and tetra-nucleotide repeats. Unit length might be another parameter affecting stability, however we cannot draw any conclusions from our own data, as the STR*Af* 3A and STR*Af* 3C markers also demonstrated the highest number of repeat units. There have been other studies though that demonstrated that unit length affects STR stability, however there is no consensus on the direction of the effect. There are studies demonstrating that shorter unit length leads to more instability (Schug et al., 1998; Vigouroux et al., 2002; O'Dushlaine and Shields, 2008; Willems et al., 2014), while other studies found the opposite (O'Dushlaine et al., 2005; Payseur et al., 2011). In the yeast *Saccharomyces cerevisiae* longer repeat units were more instable, which might be explained by an additive effect of unequal recombination, which is next to strand slippage more frequently observed in longer tandem repeats (Wierdl et al., 1997; Richard and Pâques, 2000). More research is required to demonstrate the potential role of unit length in repeat variability in fungi.

All five mutations were insertions of one repeat unit, while no deletions were found. The rate of insertions vs. deletions of tandem repeats has been studied by others. Some studies found a higher probability for an increase of the number of repeat units (Vigouroux et al., 2002; Noller et al., 2006), while others found no difference or the opposite (Vogler et al., 2006). More recent studies reported that the frequency of insertions or deletions depends on the total length of the tandem repeat, with relative longer repeats have more chance to shorten and relatively shorter repeats more likely to increase in size (Huang et al., 2002; Lai and Sun, 2003). These analyses were based on relative sizes and no absolute threshold at which an insertion is more likely than a deletion or vice versa was established. We found insertions in tri-nucleotide repeats with a length of 53–55 nucleotides, while the *A. fumigatus* genome contains many tri-nucleotide repeats with a size of >50 repeats (Levdansky et al., 2007). Based on our limited data set we cannot yet determine at which size deletions would be more likely to occur. Finally, we only found insertions of single repeat units, while other studies found that approximately half of all mutations consisted of multiple repeat changes (Bustamante et al., 2013). This might be due to the larger repeat sizes used in these studies, which would more likely lead to involving more repeats, or might be due to the fact that we analyzed every single generation, while other studies only checked a generation after ten passages, possibly leading to a bias of interpreting single additions as one multiple repeat change (Bustamante et al., 2013).

Genotyping assays based on STRs are a powerful tool for epidemiological studies on a wide range of organisms. The interpretation of data obtained with these assays requires proper understanding of the dynamics of the repeat units in STR markers. A high number of repeat units in a marker seems to have the most influence on the variability of that marker, as was also observed for the STR*Af* assay. These findings have to be taken into account when interpreting STR*Af* data during molecular epidemiological analysis.

## Author Contributions

TG and JM contributed to the acquisition, the analysis, and the interpretation of the data. Both authors drafted, reviewed, and modified the manuscript.

### Conflict of Interest Statement

JM received grants from F2G and Merck. He has been a consultant to Scynexis and Merck and received speaker's fees from Merck, United Medical, TEVA, and Gilead Sciences. The remaining author declares that the research was conducted in the absence of any commercial or financial relationships that could be construed as a potential conflict of interest.
